# Keeping Your Eye on the Rail: Gaze Behaviour of Horse Riders Approaching a Jump

**DOI:** 10.1371/journal.pone.0097345

**Published:** 2014-05-20

**Authors:** Carol Hall, Ian Varley, Rachel Kay, David Crundall

**Affiliations:** 1 School of Animal, Rural and Environmental Sciences, Nottingham Trent University, Southwell, Nottinghamshire, United Kingdom; 2 School of Science and Technology, Nottingham Trent University, Nottingham, Nottinghamshire, United Kingdom; 3 School of Social Sciences (Psychology), Nottingham Trent University, Nottingham, Nottinghamshire, United Kingdom; VU University Amsterdam, Netherlands

## Abstract

The gaze behaviour of riders during their approach to a jump was investigated using a mobile eye tracking device (ASL Mobile Eye). The timing, frequency and duration of fixations on the jump and the percentage of time when their point of gaze (POG) was located elsewhere were assessed. Fixations were identified when the POG remained on the jump for 100 ms or longer. The jumping skill of experienced but non-elite riders (n = 10) was assessed by means of a questionnaire. Their gaze behaviour was recorded as they completed a course of three identical jumps five times. The speed and timing of the approach was calculated. Gaze behaviour throughout the overall approach and during the last five strides before take-off was assessed following frame-by-frame analyses. Differences in relation to both round and jump number were found. Significantly longer was spent fixated on the jump during round 2, both during the overall approach and during the last five strides (p<0.05). Jump 1 was fixated on significantly earlier and more frequently than jump 2 or 3 (p<0.05). Significantly more errors were made with jump 3 than with jump 1 (p = 0.01) but there was no difference in errors made between rounds. Although no significant correlations between gaze behaviour and skill scores were found, the riders who scored higher for jumping skill tended to fixate on the jump earlier (p = 0.07), when the horse was further from the jump (p = 0.09) and their first fixation on the jump was of a longer duration (p = 0.06). Trials with elite riders are now needed to further identify sport-specific visual skills and their relationship with performance. Visual training should be included in preparation for equestrian sports participation, the positive impact of which has been clearly demonstrated in other sports.

## Introduction

The visual skills associated with successful performance have been identified in a number of sports and consequently have been included in preparation and training. In general these include the ability to focus on relevant information in a timely manner, with the requirements of specific sports resulting in the development of additional sport-specific visual skills. In sports such as soccer, field hockey and tennis elite athletes have been found to direct their gaze appropriately sooner, make more predictive eye movements and fixate on relevant features for longer than non-elite athletes [1], [2], [3]. In cricket the eye movements and field of gaze of batsmen associated with accurately judging the timing and placement of a ball bowled by a fast bowler were recorded using a head mounted eye camera and were found to differ according to skill level [4].

The part that visual skills play in equestrian sport has yet to be determined. In the sport of show-jumping, during the approach to the jump the rider needs to be able to predict the ‘time to contact’ (when the horse will arrive at the optimum take-off point), generally calculated by the rider in terms of ‘strides to take-off’. If this activity, which involves a high degree of spatio-temporal coordination, is likened initially to aiming at a far target then similar features of visual control as those found by Vickers in elite basketball athletes during free throws [5] may be found in elite show-jump riders. The elite basketball athletes fixated on the target for longer but had an earlier fixation offset than less-elite athletes that coincided with the movement initiation stage of the throw [5]. However, as the horse-rider combination approach the jump the target becomes closer. In other sports involving aiming at a near target, for example billiards, it has been shown that longer fixation duration is also associated with expertise, task complexity and with successful execution [6].

The term ‘quiet eye’ has been used to describe the final fixation on the target prior to the initiation of the movement needed to execute the task [5], [6]. It was proposed by Vickers that differences in visual behaviour attributable to expertise are most notable in tasks involving physical movement and the control of multiple systems in natural settings [5]. Show-jumping is undoubtedly one such scenario, in which the optimum timing, frequency and duration of fixations on the target (jump) has yet to be determined. Riders approaching a jump would be predicted to fixate on the jump until the point at which no further stride adjustment could be made. Seeing the correct stride and making appropriate stride adjustments is a skill that successful riders can apply in the final strides before take-off [7], suggesting that their fixation on the jump should continue until this point. With the use of a mobile eye tracker it was possible to monitor the timing, frequency and duration of fixations on the jump that riders make during their approach.

In an earlier study some initial pilot data was collected from two expert riders. Head orientation and gaze direction of these riders was monitored as they approached three different jump configurations. An early head-mounted eye tracker (NAC Eye Mark Recorder IV) was used and demonstrated these two expert riders consistently directed their gaze towards the upper part of the jump, regardless of its configuration [8]. Advances in eye tracking technology now allow more detailed analyses of features of sport-specific visual skills. Vickers and Adolphe [9] used a helmet mounted eye tracker to monitor fixations, tracking and quiet eye in volleyball players, using these visual parameters to assess the differences between expert and non-expert performers. More recently, glasses-mounted equipment has been developed that has the potential to provide similar information in relation to equestrian sports (for example the ASL Mobile Eye).

In equestrian sports differences in the visual behaviour of elite and non-elite athletes have to date only been demonstrated in simulated scenarios. For example, fifteen riders’ eye movements were monitored during a virtual ‘walk’ round a computer-generated show-jumping course consisting of a series of slides each depicting a different jump, and asked to describe their planning strategies. While the congruence between planned and actual visual behaviour was not significantly greater for the more expert riders when compared to the less-skilled riders different visual search strategies were apparent. The most expert riders allocated more fixations to slides than did less-skilled riders and were significantly less dependent on the overall course plan when inspecting the fences [10]. Most recently, we have demonstrated evidence for the relationship between visual skills and rider experience in a show-jumping context via the recall of visual information from static images representing approaches to jumps [11]. Relevant and irrelevant points of focus (in relation to jumping the fence) were identified in 22 photographs of show-jumps and the ability to select these from 4 alternatives was tested. Although reported riding experience did not affect overall recall, there was a significant correlation between rider experience and the ability to recall the relevant selections (but not the irrelevant ones).

In show jumping competition it is often particular jumps that are associated with errors that accrue ‘faults’ and we suggest that the position within the course, in addition to differing visual features, may also affect visual behaviour on the approach. Stachurska, Pieta and Nesteruk identified certain fence types as being problematic for show-jumping horses. The shape/configuration and colour of the fences, including individual fence position within a course of fences determined how well the horse-rider combinations jumped each fence [12]. The relative position of jumps within a course will result in variation in the potential for visual distraction, as well as differential challenges as a consequence of the previous and subsequent fences. The effect of course configuration and fence type on visual behaviour requires further investigation and may result in appropriate visual training to reduce performance problems associated with specific fence types and tracks.

The aim of this study was to use a mobile eye tracker to monitor the gaze behaviour of riders as they approached a jump. It was predicted that the timing, frequency and duration of fixations on the jump would vary with individual riders and would relate to their jumping skill. The percentage of the approach time when their position of gaze (POG) was located on the jump, ground and other locations (and when no POG was located), was also calculated. Differences in gaze behaviour in relation to the different jumps within the course (of three jumps) and in relation to round number (one to five) were assessed.

## Materials and Methods

### Ethics Statement

The study was carried out in accordance with the ethical review policy of Nottingham Trent University and approved by the School of Animal, Rural and Environmental Sciences Ethical Monitoring Group. Informed written consent for participation was obtained from participants. The horses were ridden according to NTU Equestrian Centre guidelines and were deemed fully able to carry out the exercises with ease. As such this was not a regulated procedure (Animals in Scientific Procedures Act 1986) and did not contravene the Animal Welfare Act 2006. The horses were owned by Nottingham Trent University or loaned under contract for use by the university in ridden work for any purpose. Nottingham Trent University gave permission for the use of the horses in this study.

### Participants

The gaze behaviour of ten female riders was recorded. The riders were staff at the Nottingham Trent University Equestrian Centre, each with over 15 years riding experience and aged between 22 and 45 years (mean age 32 years). Their jumping skill was assessed by self-report (see below for details). The horses ridden were regularly used in the riding school and were all used for jumping lessons for riders of various levels of ability (mean height 167.13 cm, mean age 14.8 years). Each horse was ridden by two riders.

### Design

A 5×3 within-subjects design was employed comparing rider gaze behaviour across five rounds over three fences. Features of gaze behaviour were recorded (timing, location and duration of position of gaze) and the effects of round and jump number and of the skill level of the rider on this visual behaviour were assessed. The number of errors made at each jump was also recorded.

A questionnaire was used to assess the skill level of the riders. The questionnaire was adapted from that used by Hall, Liley, Murphy and Crundall [11] and incorporated questions relating to jumping skill level (see Table 1). The questionnaire scores were then used as a measure of rider skill.

**Table 1:** Questionnaire used for rider self-reported assessment of competitive experience.

Questions and response scales					
**Question 1**	I could complete a course of show jumps at a height of:				
*Response options*	60 cm	75 cm	90 cm	105 cm	120 cm
*Score*	**1**	**2**	**3**	**4**	**5**
**Question 2**	Have you ridden competitively in a discipline involving show jumping (either currently or in the past)?				
*Response options*	Yes (currently)	Yes (in the past)	No		
*Score*	**3**	**2**	**1**		
**Question 3**	If you answered yes to Question 2, at what level have you competed?				
*Response options*	Pre-novice	Novice	Intermediate	Advanced	
*Score*	**1**	**2**	**3**	**4**	
**Question 4**	At what national/international level?				
*Response options*	Local	County	Regional	National	International
*Score*	**1**	**2**	**3**	**4**	**5**

Table showing the questions, optional responses and associated scores used to calculate the self-reported jumping skill of the riders.

### Test Area and Materials

**Figure 1:**
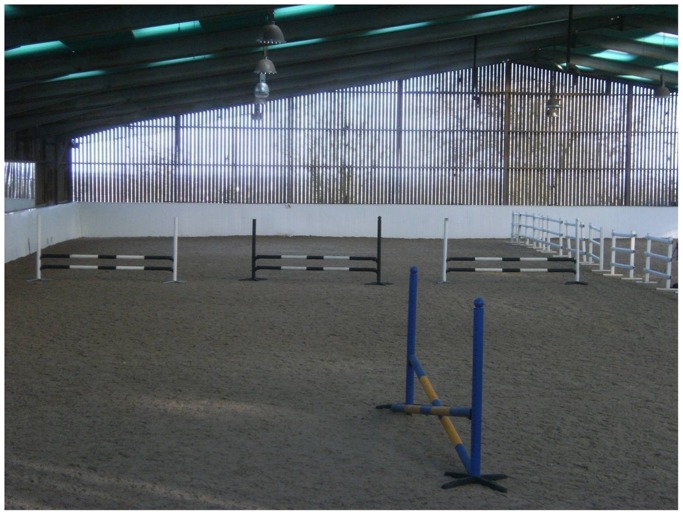
Test arena, course of 3 trial jumps and practice jump. The test area, three trial jumps and the practice jump are shown. The blue and yellow jump in the foreground was used as the practice jump. The course was approached from this end with the jump on the left being jumped first, followed by a right turn across the diagonal (jump 2) and then a left turn to approach jump 3 (adjacent to the white barriers).

The course of jumps was set up at in an indoor riding arena (40 m×55 m) with a silica sand wax and fibre mix surface (Softrack). The course consisted of three almost identical jumps (the only difference was that jump 1 and 3 had white supporting stands and jump 2 had black supporting stands). The course and practice jump were situated in one side of this arena in an area of 20 m×55 m which had been sectioned off by means of a central white plastic barrier 1.20 m in height. The jumps conformed to BSJA specifications and were produced by Jump 4 Joy. The jump cups were (FEI) approved safety cups also made by Jump 4 Joy. Each jump was an upright consisting of two black and white striped rails (straight poles resting on jump cups at 0.40 m and 0.70 m from the ground respectively), the overall height of which was 0.725 m, the overall width 4.00 m. The first and third jumps were placed at 90° to the long side and central barrier of the arena respectively. The second jump was placed across the diagonal in between the other two. All three jumps were situated in a line across the arena, 24 m from the end wall. The riders approached the first jump on the right rein, turned right to jump the second jump and then left over the third. The practice fence (a cross pole with a central height of 0.45 m) was positioned centrally and in parallel with the long side/central barrier at the opposite end of the arena to the test course. The test area, course of three jumps and the practice jump are shown in Figure 1.

### Apparatus

**Figure 2:**
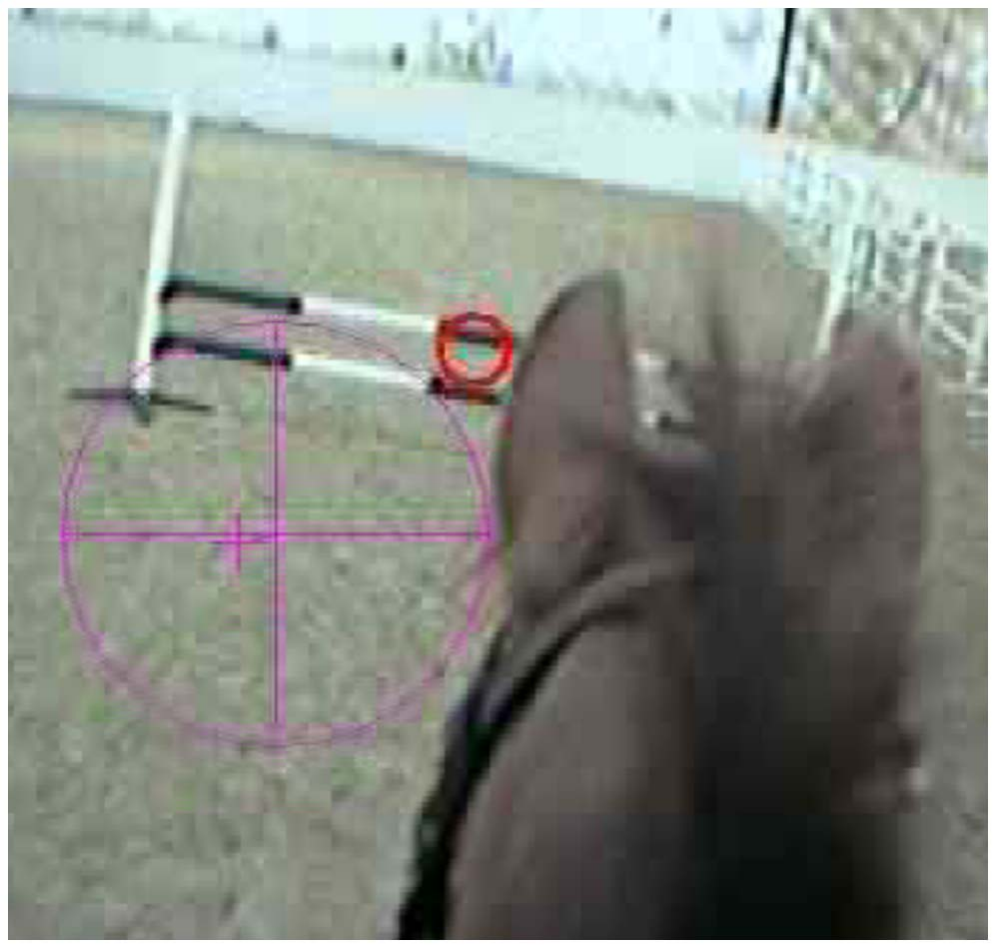
Frame showing POG cursor (red circle) and pupil indicator (purple circle) on jump approach. Frame taken from the ASL Mobile Eye recording showing POG (red circle) and pupil indicator (purple circle) as a rider approaches a jump. The image was taken from footage from the eye tracker and consequently the resolution of the image is limited. The frame provides an example of the information recorded by the equipment and the gaze behaviour measures were calculated using frame by frame analyses.

A mobile eye tracking device (Applied Science Laboratories (ASL), Bedford, USA: Mobile Eye), was used to monitor the gaze behaviour of the riders. This required the rider to wear a spectacle-mounted unit (SMU: consisting of a scene camera and an eye camera) which was attached via the recorder mounted unit (RMU) to a digital video cassette recorder (DVCR) housed in a backpack worn by the rider. The ASL Mobile Eye recorded data at 60 Hz by interleaving images taken from the two cameras. The eye camera recorded the eye being tracked whilst the scene camera recorded the environment being observed by the wearer. Both image streams were recorded on the same digital videotape medium by alternating frames and position of gaze (POG) was shown by a red cursor and was recorded every frame of video or 33.33 milliseconds. A purple circle appeared within the frame and indicated that the pupil had been identified [13]. See Figure 2 for an example of scene camera/POG cursor as recorded during these trials (the resolution of this image is that recorded by the scene camera and cannot be improved).

The Mobile Eye used a technique of eye tracking known as dark pupil tracking, which uses the relationship between two eye features, the pupil and a reflection from the cornea to compute gaze within a scene. Calibration of the equipment was carried out to determine the POG of each individual. The POG was calibrated every time the Mobile Eye was fitted to ensure that small differences in spectacle position did not affect the recording [13]. The equipment and calibration was checked repeatedly throughout the trials because of the movement associated with riding a horse over jumps and with mounting initially. An x-point calibration was performed with calibration points (N = 10) located at varying heights and planes of motion. The calibration of the spot cluster and pupil display settings of the equipment were checked before the rider mounted, then once the rider had mounted, after the warm-up period and practice fence and after each round within the trial session.

### Procedure

Within the test session the rider was fitted with the eye tracking device before mounting the horse and the initial POG calibration was carried out. The rider then walked the course of jumps while wearing the equipment to ensure that they were not adversely affected by it. After mounting the horse the equipment was checked to ensure that the eye and scene cameras were in the correct position and a second POG calibration was carried out. The rider then prepared the horse on the flat before jumping the practice fence twice in each direction. The eye tracking equipment was then re-checked before the rider jumped the test course (test course jumps shown in Figure 1). The course was jumped five times by each rider, who returned to have the equipment re-checked in between each round. Each rider jumped a total of 15 jumps within the session. Jumping errors were recorded and a score allocated (similar to the faults system in show jumping). If the pole was knocked but did not fall a score of 2 faults was allocated; a pole that was knocked down scored 4 faults. The maximum error that could be scored was 60 points. Total scores for each rider were calculated for each jump number and for each round number for comparison with gaze data.

### Gaze Data Collection and Analysis

EyeVision data processing software was used to calibrate POG from the recorded data and the data stream for each of the ten riders was recorded from the DVCR using EyeVision for identification of POG. The location of POG was recorded for each frame as being either on the **jump** (defined as part or all of the gaze cursor being located on the jump or on the area between the two poles), on the **ground** (any area of the ground in front, to the side of or beyond the jump), or on **other** (which included all other locations within the scene, for example, the horse, arena wall, mirror, people, gallery and other jumps). Frames in which no gaze cursor was visible were recorded as **no eye**. In order to capture all of the visual behavior associated with the jump being approached POG location was recorded starting with the frame in which the jump in question was first visible on the scene camera and finishing when the jump pole disappeared from view. Following frame-by-frame analysis the number of consecutive locations of the POG on the **jump** was recorded. Minimum fixation duration was 100 ms (≥3 frames) as specified by Vickers and Adolphe [9] and defined as stabilization of the gaze on the jump. The number and duration of each fixation was calculated for each rider/round/jump combination. The timing of the start of the first fixation on the jump, the longest fixation and the offset of the final fixation on the jump was calculated. To take account of potential differences in the speed of approach, the number of canter strides taken by the horse during the approach time was counted from the scene camera footage (to the nearest stride) and the speed of each approach calculated from this as the number of strides per second. If the approach time had included trot then the speed was calculated from the time when the first canter stride occurred. This speed was used to calculate the number of strides from the jump that the first, longest and the offset of the final fixation on the jump occurred.

The total duration of all fixations on the jump was assessed and calculated as a percentage of the approach time. The percentage of approach time when the POG was located on the jump for <100 ms (saccades) was calculated. The percentage of approach time where the POG was located on the **ground,** **other** or **no eye** was recorded was also calculated.

The minimum number of strides recorded for any rider during the approach was identified as being five strides. To allow consistent comparison of the riders, regardless of their speed, their gaze behaviour during the last five strides of the approach to the jump was also analysed. The number and mean duration of fixations during the last five strides of the approach were calculated. If the first stride commenced mid fixation on the jump this fixation was included in the number and the duration was included in the calculation of the mean fixation duration. However, only frames within the stride window were included in the calculation of the percentage of time spent fixated on the **jump**. Percentages of time spent with POG on the **ground**, **other** and **no eye** were also calculated for the last five strides.

The timing and duration of the first, longest and final fixation on the jump were calculated as a means of further assessing the visual strategy used by riders during the approach to the jump. If there was more than one fixation at the longest duration then the one closest to the take-off point was counted.

When the percentage of missing data (no eye recorded) was calculated it was found that during the overall approach no eye was recorded for an average of 21.39±12.80% of the time and during the last five strides for an average of 20.05±4.65%. It could not be assumed that when no eye was recorded the rider was not looking at the jump and subsequently correlations with rider skill that included only trials where no eye was recorded for ≤20% of the approach time were investigated. This resulted in between 0 and 15 trials being included for each rider for the overall approach (a total of 84; 56% of all trials). For the analyses for the last five strides this also resulted in between 0 and 15 trials being included for each rider (a total of 88; 58.67%). No data from the lowest scoring rider could subsequently be included in either of the analyses.

The distribution of the data was assessed for normality using the Kolmogorov Smirnov test and subsequently parametric and non-parametric analyses were conducted. Correlations between rider skill level, errors made and the measures of gaze behaviour were investigated (Spearman’s Rank Order Correlation).

The effect of round and jump number on the speed and time of the approach, and on each measure of gaze behaviour during the overall approach and during the last five strides of the approach to the jump was assessed (two-way within subjects ANOVA). Pair-wise comparisons adjusted for multiple comparisons (Bonferroni) were subsequently conducted. The effect of round and jump number on jump errors made was also assessed (Friedman’s test) with subsequent pair-wise comparisons (Wilcoxon test).

Analyses were carried out using SPSS version 21.

## Results

The overall approach to the jump, from the first appearance of the jump on the scene camera to the disappearance of the pole, took between 2.63–9 seconds (mean 5.66±0.87 s). The number of strides within this overall approach time ranged from 5–15 strides (mean 9.87±1.44 strides). The speed of the approach ranged from 1.32–2.07 strides/second (mean 1.75±0.07 strides/s).

### Location of Position of Gaze

During the **overall approach** to the jump the percentage of time during which the rider was fixated on the jump ranged from 0–63.89% (mean 19.78±12.69%). The percentage of the approach time when the POG was recorded on the ground ranged from 7.39–51.72% (mean 30.21±7.41%). The percentage of the approach time when the POG was recorded on other ranged from 0–57.93% (mean 23.72±10.49%). The percentage of the approach time when the POG was not recorded (no eye) ranged from 0–73.39% (mean 21.39±12.89%).

During the **last five strides** of the approach the rider was fixated on the jump for a slightly higher percentage of the time, from 0–77.07% (mean 23.29±16.02%). The percentage of time when the POG was located on the ground was slightly lower, ranging from 0–67.46% (mean 26.55±10.01%). The percentage of time when the POG was located on other was comparable with that of the overall approach, ranging from 0–57.93% (mean 23.31±10.03%). The percentage of time when no eye was recorded was also similar to the overall approach, ranging from 0–82.20% (mean 20.05±16.47%).

See Table 2 for the location of POG for the individual riders and their associated skill scores during the overall approach and during the last five strides of the approach.

**Table 2:** Mean percentage (±standard deviation) of approach time that POG was recorded on each location during the total approach time and during the final five strides^1^.

	Mean percentage of approach time that POG recorded on different locations (±standard deviation). Percentage on jump relates to fixations only (POG ≥100 ms)							
	Total approach to jump				Final five strides before take-off			
Rider skill score	Mean % approachtime fixatedon jump	Mean % approachtime POG onground	Mean % approachtime POGon other	Mean % approachtime no eyerecorded	Mean % approachtime fixatedon jump	Mean % approachtime POG onground	Mean % approachtime POGon other	Mean % approachtime no eyerecorded
**21**	26.00 (±7.73)	36.25 (±6.39)	18.17 (±7.90)	16.02 (±6.66)	25.29 (±10.75)	35.52 (±11.81)	24.63 (±15.13)	12.36 (±8.04)
**14**	35.48 (±15.41)	24.47 (±10.90)	19.48 (±15.68)	17.10(±8.32)	48.63 (±17.94)	32.51 (±15.38)	6.29 (±6.05)	10.36 (±9.63)
**14**	12.27 (±8.56)	38.89 (±6.36)	21.06 (±5.78)	21.76 (±4.16)	9.10 (±7.90)	32.30 (±5.74)	30.82 (±10.40)	21.61 (±7.68)
**11**	37.08 (±11.79)	32.65 (±6.29)	19.76 (±6.60)	5.66 (±3.54)	33.41 (±19.30)	31.17 (±15.14)	29.09 (±15.58)	5.45 (±5.12)
**11**	30.65 (±9.89)	39.21 (±8.11)	17.97 (±7.10)	8.63 (±12.54)	43.29 (±15.21)	19.81 (±8.94)	26.78 (±7.99)	7.22 (±14.53)
**10**	6.50 (±4.83)	26.03 (±13.20)	40.73(±11.84)	21.37 (±7.15)	8.37 (±8.60)	33.44 (±8.35)	27.16 (±7.47)	24.55 (±12.00)
**10**	3.34 (±5.49)	32.63 (±10.44)	17.04 (±10.71)	42.91 (±14.12)	4.60 (±5.66)	28.36 (±10.98)	19.45 (±16.36)	42.38 (±17.40)
**9**	26.15 (±10.57)	32.57 (±6.40)	12.03 (±6.63)	23.56 (±10.93)	34.50 (±10.72)	33.09 (±8.36)	9.05 (±4.77)	17.39 (±7.17)
**8**	13.16 (±6.39)	22.24 (±6.25)	43.81 (±9.56)	13.48 (±7.17)	17.50 (±9.55)	6.38 (±3.20)	39.71 (±7.67)	5.13 (±4.19)
**5**	7.13 (±4.62)	17.15 (±5.96)	27.14 (±9.47)	43.44 (±11.05)	8.19 (±6.39)	12.90 (±5.67)	20.07 (±10.30)	54.08 (±16.39)
**All riders**	**19.78 (±12.69)**	**30.21 (±7.41)**	**23.72 (±10.49)**	**21.39 (±12.80)**	**23.29 (±16.02)**	**26.55 (±10.01)**	**23.31 (±10.03)**	**20.05 (±16.47)**

1 The % time for the jump relates to fixations only and the % not accounted for relates to POG located on the jump for <100 ms.

Riders listed in descending order according to skill score.

### Timing, Frequency and Duration of Fixations on the Jump

The first fixation on the jump occurred at between 12.2 and 0.23 seconds before take-off (mean 4.36±1.25 s), between17 and 0.5 strides before take-off (mean 6.98±1.79 strides). Three riders did not fixate at all on the jump during one (n = 2 riders) and seven (n = 1 rider) jump approaches. Between 0 and 13 fixations on the jump were made during the overall approach (mean 4.91±2.07). During the last five strides between 0 and 7 fixations on the jump were made (mean 2.79±1.16). The duration of the fixations on the jump ranged from 99.99–2933.04 milliseconds in the overall approach (mean 226.60±121.82 ms), during the last five strides (mean 250.06±154.00 ms), for the first fixations on the jump (mean 181.34±76.65 ms) and for the final fixations on the jump (mean 240.42±154.80 ms). The offset of the final fixation occurred at between 5.16 and 0.07 seconds before take-off (mean 1.23±0.43 s); at between 8.80 and 0.12 strides before take-off (so approximately at the point of take-off) (mean 2.20±0.70 strides). The mean duration of the longest fixation was 378.70±262.30 ms and this occurred at between 9.09 and 0.23 seconds (mean 2.49±0.69 s) and between 11.99 and 0.45 strides (mean 4.35±1.17 strides) before take-off.

See Table 3 for the timing, frequency and duration of fixations on the jump for individual riders during the overall approach and during the final five strides.

**Table 3:** Duration, timing and frequency of fixations on the jump during the approach (riders listed in descending order according to skill score).

	Fixations on the jump during the approach (mean values ± standard deviation)									
	Overall approach		Last five strides		First fixation		Longest fixation		Final fixation	
RiderSkillscore	Mean number	Meanduration(ms)	Mean number	MeanDuration(ms)	Time (s) and strides(str) before take off	MeanDuration(ms)	Time (s) and strides(str) before take off	MeanDuration(ms)	Offset (s) and strides(str) before take off	MeanDuration(ms)
**21**	6.60 (±1.99)	244.38 (±58.40)	3.33 (±1.11)	254.86 (±80.70)	4.95 (±1.22)s 8.37 (±2.16)str	199.98 (±84.51)	2.68 (±1.06)s 4.54 (±1.82)str	415.51 (±80.53)	1.46 (±0.45)s 2.48 (±0.78)str	264.42 (±153.50)
**14**	6.87 (±2.95)	522.62 (±684.39)	3.80 (±1.66)	602.08 (±703.31)	6.47 (±3.63)s 7.80 (±1.94)str	382.18 (±717.16)	3.34 (±2.57)s 5.86 (±4.89)str	991.01 (±741.70)	0.86 (±0.37)s 1.49 (±0.62)str	628.83 (±868.40)
**14**	4.87 (±2.83)	152.62 (±50.60)	1.93 (±1.49)	141.47 (±42.34)	4.96 (±1.33)s 8.48 (±2.19)str	126.18 (±39.61)	3.25 (±1.23)s 5.54 (±2.06)str	238.07 (±110.05)	1.84 (±0.86)s 3.14 (±1.46)str	130.94 (±38.03)
**11**	7.67 (±1.59)	288.71 (±69.49)	3.00 (±1.46)	390.16 (±131.96)	5.26 (±1.23)s 9.71 (±2.25)str	202.20 (±98.77)	3.03 (±0.72)s 5.60 (±1.36)str	475.51 (±143.89)	1.36 (±0.79)s 2.53 (±1.51)str	284.42 (±166.11)
**11**	5.53 (±2.00)	287.93 (±87.86)	3.93 (±0.88)	328.41 (±107.67)	3.68 (±0.64)s 6.39 (±1.11)str	168.87 (±64.81)	1.84 (±0.50)s 3.20 (±0.90)str	584.39 (±266.90)	0.75 (±0.33)s 1.31 (±0.56)str	295.53 (±228.45)
**10**	2.53 (±1.51)	132.95 (±35.40)	1.47 (±1.25)	137.25 (±40.59)	3.14 (±1.15)s 5.88 (±2.09)str	128.88 (±58.91)	2.41 (±0.97)s 4.49 (±1.73)str	162.21 (±65.30)	1.74 (±1.01)s 3.26 (±1.83)str	131.10 (±44.48)
**10**	1.27 (±1.98)	113.88 (±15.43)	1.07 (±1.28)	113.88 (±15.43)	1.90 (±1.34)s 3.49 (±2.36)str	129.15 (±33.03)	1.46 (±0.87)s 2.70 (±1.51)str	129.15 (±33.03)	0.97 (±0.60)s 1.78 (±1.04)str	112.49 (±24.80)
**9**	5.47 (±2.33)	216.11 (±33.48)	4.40 (±1.24)	234.16 (±40.88)	4.67 (±3.77)s 5.42 (±1.95)str	175.54 (±54.14)	1.64 (±0.77)s 2.83 (±1.38)str	386.63 (±144.63)	0.66 (±0.24)s 1.64 (±0.49)str	266.64 (±140.85)
**8**	5.47 (±2.64)	142.91 (±27.45)	3.27 (±1.49)	154.80 (±40.01)	4.36 (±1.34)s 7.59 (±2.40)str	126.65 (±40.23)	2.17 (±0.89)s 3.78 (±1.65)str	202.20 (±98.23)	1.00 (±0.28)s 1.74 (±0.48)str	142.21 (±62.31)
**5**	2.87 (±1.77)	163.91 (±60.65)	1.73 (±1.28)	143.50 (±29.28)	4.20 (±2.29)s 6.72 (±3.08)str	173.79 (±74.16)	3.10 (±2.32)s 4.98 (±3.34)str	202.36 (±98.23)	1.60 (±1.23)s 2.67 (±2.12)str	147.60 (±36.31)
**All riders**	4.91 (±2.07)	226.60 (±121.82)	2.79 (±0.22)	250.06 (±207.13)	4.36 (±1.25)s 6.98 (±0.49)str	181.34 (±208.56)	2.49 (±0.69)s 4.35 (±1.18)str	378.70 (±209.61)	1.23 (±0.43)s 2.20 (±0.70)str	240.42 (±252.90)

### Variation in Approach and Gaze Behaviour in Relation to Round/Jump Number

The speed of the approach to the jump (mean 1.75±0.07 strides/s) did not vary with round or jump number (two-way within subjects ANOVA: round F(4,36) = 1.14, p = 0.354; jump F(2,18) = 1.48, p = 0.255; round/jump interaction F(8,72) = 1.60, p = 0.14). There was a significant main effect of jump number on the time of the **overall** approach (F(2,18) = 16.00, p<0.001) with the approach to jump 1 (mean 6.61±0.30 s) being significantly longer than jump 2 (mean 5.43±0.29 s) (p = 0.007) or jump 3 (mean 4.93±0.22 s) (p = 0.001). There was no significant difference between the approach time to jump 2 and jump 3 (p = 0.48). No effect of round number on the approach time was found (F(4,36) = 1.859, p = 0.139). There was no significant effect of round or jump number on the approach time for the last **five strides** (two-way within subjects ANOVA: round F(4,36) = 0.96, p = 0.44; jump F(2,18) = 1.54, p = 0.24; round/jump interaction F(8,72) = 1.57, p = 0.15).

The total number of errors made varied significantly with jump number (Friedman: X^2^ (2, n = 10) = 6.75, p = 0.034). More errors were made with jump 3 (3.6±1.02) than with jump 1 (1.2±0.44), (Wilcoxon: Z = 2.46, p = 0.014). No difference between the errors made with jump 2 (1.6±0.72) and either of the other jumps was found. No significant effect of round on the errors made was found (Friedman: X^2^ (4, n = 10) = 5.83, p = 0.21).

There were two features of gaze behaviour that varied significantly with round number. During the overall approach the percentage of time the riders spent fixated on the jump varied with round number (F(4,36) = 3.051, p = 0.029) and was greatest during round 2. When the gaze behaviour during the last five strides was assessed there was also a significant variation in the percentage of time spent fixated on the jump during the different rounds (F(4,36) = 3.617, p = 0.014) with the highest percentage occurring in round 2. No effect of jump number on the percentage of time spent fixated on the jump was found during the overall approach (F(2,18) = 0.049, p = 0.953) or during the last five strides (F(2,18) = 0.342, p = 0.715). During the overall approach the percentage of time when the POG was located on other also varied significantly with the round number (F(4,36) = 2.903, p = 0.035) being highest during round 5. No variation in relation to jump number was found (F(2,18) = 1.040, p = 0.374). See Table 4. No significant variation in any other aspect of gaze behaviour was found in relation to round number.

**Table 4:** Significant variation in gaze behaviour in relation to round number^1^.

	Round 1	Round 2	Round 3	Round 4	Round 5	Significance value (p)	Effect size (partial eta squared)
**Overall approach**							
% approach fixated on **jump**	21.95±5.44	24.29±4.91	19.81±3.56*	16.05±3.44*	16.79±4.25	0.029	0.253
% approach POG on **other**	21.27±2.98	22.04±3.11	25.20±3.72	22.94±3.59	27.14±4.22	0.035	0.244
**Last five strides**							
% approach fixated on **jump**	24.72±5.75	30.59±6.53*	22.47±4.92	18.81±4.83*	19.84±5.41	0.014	0.287

1 Mean percentages ±standard error shown for each round.

Significant difference between rounds *p<0.05.

There were three features of gaze behaviour that varied significantly with jump number. During the overall approach the timing of the first fixation varied significantly with jump number (F(2,12) = 7.983, p = 0.006) being earliest for jump 1. No difference in the timing of the first fixation was found in relation to round number (F(4,24) = 1.066, p = 0.395). The number of fixations during the overall approach varied significantly in relation to jump number (F(2,18) = 10.869, p = 0.001) with the most fixations occurring with jump 1. No effect of round number on the number of fixations was found in relation to round number (F(4,36) = 1.734, p = 0.164). The timing of the longest fixation also varied significantly with jump number (F(2,12) = 7.762, p = 0.007) with the earliest fixation occurring on jump 1. No effect of round number on the timing of the longest fixation was found (F(4,24) = 0.637, p = 0.641). See Table 5. No significant variation in any other aspect of gaze behaviour was found in relation to jump number.

**Table 5:** Significant variation in gaze behaviour in relation to jump number^1^.

	Jump 1	Jump 2	Jump 3	Significancevalue (p)	Effect size (partialeta squared)
**Overall approach**					
**Timing** of **first** fixation on **jump** (seconds before take-off)^2^	6.25±0.86 s	4.14±0.37 s	3.56±0.35 s	0.006	0.571
**Number** of fixations on **jump**	6.28±0.76*,**	4.92±0.78*	3.88±0.63**	0.001	0.547
**Timing** of **longest** fixation on **jump** (seconds before take-off)^2^	2.98±0.36 s*	2.29±0.13 s	2.07±0.27*	0.007	0.564

1 Mean values±standard error shown for each jump.

2 Mean values derived from the seven riders who fixated on all jumps.

Significant difference between jumps *p<0.05; **p<0.01.

### Correlation between Gaze Behaviour and the Reported Jumping Skill of the Rider

There was no correlation between reported jumping skill and either the total time of the overall approach (Spearman’s rho = 0.36, n = 10, p = 0.31) or the time of the last five strides (Spearman’s rho = −0.01, n = 10, p = 0.97).

The following correlation results include only trials where no eye was recorded for ≤20% of the approach time. Although no correlation was found between jumping skill and the percentage of no eye recorded during the overall approach (Spearman’s rho = −0.41, n = 10, p = 0.24) or during the last five strides (Spearman’s rho = −0.25, n = 10, p = 0.50), the location of the POG could not be recorded.

### i) Location of POG

No significant correlation between rider skill and the percentage of time the POG was located on the jump, ground or other was found during the overall approach or during the last five strides.

### ii) Timing, Duration and Frequency of Fixations on the Jump

When only those trials where the percentage of missing data was ≤20 were considered, no significant correlations between rider skill scores and gaze behaviour were found. However, there was a tendency for those riders with higher jumping skill scores to fixate on the jump earlier (Spearman’s rho = 0.63, n = 9, p = 0.07), when the horse was further from the jump (Spearman’s rho = 0.59, n = 9, p = 0.09) and for the first fixation on the jump to be of longer duration (Spearman’s rho = 0.64, n = 9, p = 0.06). It was found that the earlier the jump was fixated on the more frequently it was fixated on during the approach (Spearman’s rho = 0.70, n = 9, p = 0.04). Also, the longer the duration of the first fixation on the jump, the more frequent the fixations (Spearman’s rho = 0.67, n = 9, p = 0.05).

## Discussion

The results of this study suggest that there are a number of factors that affect the gaze behaviour of riders as they approach a jump, even in a non-competitive situation and when the jumps in question are uniform in appearance and relatively unchallenging in size. Some association was also found between certain factors and the jumping errors made. Differences in gaze behaviour were found during the five rounds. The percentage of the approach time spent fixated on the jump was greatest during round 2, particularly evident during the last five strides. This percentage was least during round 4. The percentage of time spent with the POG recorded on other was least during round 1 and greatest during round 5. See Table 4. It appears that fixations on the jump decline with repetition and that the POG is directed elsewhere. This did not have any deleterious consequences for the clearance of the relatively small jumps in this study but may cause a decline in performance when larger jumps are involved. In show jumping competitions it is likely that any repeated courses would have a time element involved which may serve to counteract any changes in visual behaviour that could occur as a consequence of this repetition. This effect should be considered during training however, where repeated jumping of the same obstacle may detract from performance.

Despite the three jumps being almost identical more errors were made with jump 3 than with jump 1. The position in the course affected how well the approach to the jump was visually planned and more errors occurred at the last jump. The timing of the first fixation and the number of fixations on the jump appeared to relate to how easily it was jumped. As all three jumps were almost identical in appearance and construction it is likely that the differences found in gaze behaviour were a consequence of differences in the location of the jumps within the test area and their sequential position within the course. In order to guide the horse towards the jump the rider needs to direct their gaze to enable visual planning of the approach. As was found in the current study, visual planning is likely to be most advanced for the first in a series of jumps and also to include fixations on the other jumps prior to the approach in order to plan the route around the whole course. Once the approach to the first jump has commenced, subsequent jumps were not fixated on until the horse had taken off over the first jump. In the case of one rider it was at this point that jump 2 was fixated on. The optimum timing of this first fixation will depend upon the course design, proximity and construction of the related fences. In the current study the height of the jumps did not pose any real challenge to any of the horses or riders, the distances between them did not require tight turns to be made and there was no speed element involved. The tracks between jump 1 and jump 2 and between jump 2 and jump 3 were comparable. However, jump 3 was still fixated upon later than jump 2, even if not significantly so. Further work is required to determine whether the later timing of the first fixation on the last jump occurs in competitive jumping and also whether this is affected by the total number of jumps within a course (generally greater than the three used in this study). Also, whether there is a general reduction in the timing of the first fixation on the jump throughout a course as indicated by our findings, or whether these times are maintained within a competitive situation, when avoidance of jump errors is more important.

The position within the test area may also have affected gaze behaviour. The approach to jump 1 was along the arena wall and there was a relatively long, uninterrupted straight approach to this jump. There was some evidence of riders fixating on the wall mirrors but potentially less distractions than alongside jump 3. Jump 3 was located close to the central barrier and although the approach was straight, the horse/rider may have been distracted by activity in the other half of the arena. Such distractions are inevitable in competitive scenarios and may well impact on visual behaviour and associated performance. A greater number of fixations were recorded for jump 1 than for jump 3, although the mean duration of these fixations did not differ.

The effect of skill level on gaze behaviour could only be assessed for a limited number of trials as a consequence of the high proportion of missing data. The percentage of the approach time when no eye was recorded could be accounted for in a number of ways. The percentage of time when the gaze cursor was absent from the scene footage (recorded as no eye) varied from 0–73.39% (mean 21.39±12.89%) during the overall approach to the jump and 0–82.20% (mean 20.05±16.47%) during the last five strides. No gaze cursor appearing on the scene footage for approximately 5–10 consecutive frames could potentially be accounted for by the wearer blinking [13]. Longer periods require further explanation. When the coordinate data recorded by the EyeVision software was compared with the scene footage it was found that in some instances the coordinates recorded were outside the range of the scene camera (768×576 pixels) and the gaze cursor could not therefore be displayed (despite the POG having been located). The field of view of the scene camera (60° horizontally and 40° vertically) was compared with the field of view of the wearer (estimated in a post hoc trial as being approximately 90° horizontally and 60° vertically with 37° of the latter in the lower visual field). The discrepancy between the two suggested that when the wearer looks out of the corner of their eye (without moving their head) it may fall out of the field of view of the scene camera. An additional explanation for the missing gaze cursor is that this occurs when the rider is looking down, which is a common fault in riders (personal communication). The position of the eye during frames when no gaze cursor was recorded was assessed from the eye camera footage and it was found that these often coincided with the eye being half closed and the POG directed down towards the ground. When recording POG for each frame it was noted that often in the frames immediately prior to the disappearance of the gaze cursor the POG would be located towards the periphery of the scene, including the bottom edge. During some periods of no eye it was common for the scene camera to be showing the horse’s neck and shoulder and the ground, suggesting that the rider was indeed looking down. The percentage of time when the gaze cursor was missing varied with individual riders and may well relate to differences in their visual behaviour but was not found to relate to skill level. However, it was noted that the number of errors made were greater when higher percentages of no eye was recorded during the last five strides of the approach (Spearman’s rho = 0.57, n = 10, p = 0.085) although not significantly so. The impact of this on performance requires further investigation.

The riders in the present study were all experienced but non-elite riders with varying levels of reported jumping skill. As shown in Tables 2 and 3 the riders also varied considerably in terms of their visual behaviour. In other sports elite athletes have been shown to direct their gaze on relevant visual information sooner and fixate on relevant features for longer than non-elite athletes [1], [2], [3]. Some comparable features were found in the more skilful riders in the present study. Their first fixation on the jump tended to be for longer and the longest fixation during their approach tended to be earlier both in terms of time and strides to take-off.

In other sports the duration and timing of each fixation has been associated with skill level with elite athletes exhibiting a period of ‘quiet eye’ (a fixation or tracking gaze that is located on a specific location for a minimum period of 100 ms, the onset of which occurs prior to the final movement needed to execute the task) [5], [6]. In order to assess whether (and if so, when) a comparable period of gaze fixation on the jump occurs during the approach the timing and duration of the longest fixation was identified. The start of the longest fixation varied from approximately 1.5–3.5 seconds before take-off (2.5–5 strides from the jump). Although no consistent timing for this longest fixation was found, the rider who was recorded as having the longest fixation duration (rider with the equal second highest reported skill level of 14) started this fixation at approximately 3.5 seconds (almost 6 strides) from the jump. The same rider also had the longest duration of final fixation on the jump, with an offset of less than 1 second before take-off. See Table 3. Although the rider with a skill score of 21 had greater overall jumping experience this related more to the equestrian discipline of eventing, whereas the other rider was predominantly a show-jump rider. The visual skills associated with show-jumping (rather than with jumping *per se*) may be specific enough to account for these differences and further investigation is required. There is the potential for improving the performance of event riders in the show-jumping phase of this discipline by identifying these skills and implementing the findings within training.

The point at which no further adjustment can be made to the approach to the jump would be expected to correspond with the offset of the longest fixation on the jump. The lack of consistency in the timing of this within the current study could relate to the varying ability of the riders to influence the stride of the horse on the approach. It was noted by Smart that too many people try to see a stride too far away from the jump (five or six strides away) and that top horse/rider combinations can turn to large fences from only one or two strides away and still successfully clear the jump [7]. This indicates that the fixation on the jump should continue until the point of take-off or at least until the final stride. The jumps within this study were relatively small and could be cleared successfully by the horse/rider combinations even if the striding on the approach did not result in take-off at the optimum point. In a more challenging task, as has been shown in other sport [6], visual skills may be more crucial to successful completion of the task of clearing the jump. To identify whether there is an optimum visual strategy in the sport of show-jumping the timing and duration of fixations during the approach to larger, more challenging jumps requires evaluation. In order to assess the impact of gaze behaviour on the negotiation of these jumps it would be valuable to simultaneously monitor the approach gait and take-off point of the horse, as well as the success or not of clearance.

When gaze behaviour during the overall approach was compared with that during the last five strides some differences in gaze behaviour were found (see Tables 2 and 3). Previous work identified the point of focus of experienced jump riders as being the upper part of obstacles with a fixed head position being maintained on the approach, although only the last two seconds of their approach was reported [8]. Visual planning was found to occur well in advance of the two seconds prior to take off and earlier fixations on the jump were associated with greater jumping skill. This measure would be influenced by the track taken by the rider and the speed of the approach, although no significant differences in speed or times were found. In particular, the first fixation on jump three could have been influenced by whether the rider went on the inside or outside of the practice fence. In general, the least skilful riders took the outer track, with the more skilful riders approaching the third jump from inside the practice jump. The latter still tended to fixate on the jump earlier, regardless of track. Although the timing of the first fixation on the jump will depend upon the space and distance between jumps it was found that this tended to occur earlier in more skilful riders.

The riders were found to have their POG located on the ground for up to 40% of the approach time. This may have related to looking for the optimum take-off point. Previous work has indicated that peripheral visual information plays only a minor role in the control of the locomotion of the horse by the rider [8] but the use of other visual cues has yet to be determined. During the current study in addition to the fixations on the jump and ground, POG was also located on other environmental features such as the wall of the arena, other horses and observers. The location of the POG on the wall in front of the jump may relate to the jump approach. No differences were found in relation to skill and the value of this aspect of gaze behaviour also requires further investigation. Some features of gaze behaviour could not be related to the approach to the jump and may have constituted distraction from the task, e.g. POG on other horses, people. In some cases these occurred for less than 100 ms and were not long enough for the rider to be aware of this distraction. It is likely that stimuli within a jumping arena and its surroundings will distract riders and if such distractions are found to detract from performance then further training aimed at improving concentration could have a beneficial effect on performance.

In order to fully ascertain the visual skills associated with the equestrian sport of show jumping, detailed analysis of the visual behaviour of a number of elite athletes is now required. The results of the current study have demonstrated that visual skills are apparent in equestrian sport comparable with those found in other sports. The identification of elite visual skills will enable equestrian sport to utilize this information in training and preparation for competitive events, with potential benefits for enhancing performance. Variation in gaze behaviour in relation to the position of the jump within the course may have been the result of the specific course set-up used in this study, but could also be a feature common to jumping sequences of jumps. There may be a relationship between the gaze behaviour and errors made but this needs further exploration before conclusions can be drawn. However, visual skills are undoubtedly associated with skill and success in equestrian sport. Recent advances in mobile eye tracking technology will facilitate further identification of the precise nature of these sport-specific visual skills and their subsequent application in performance enhancement.

## Conclusion

This study demonstrates the use of mobile eye-tracking equipment to monitor gaze behaviour in a real show-jumping scenario. As in other sports there was some evidence of a relationship between jumping skill and gaze behaviour during the approach to jumps. Confirmation is now required by monitoring gaze behaviour in elite equestrian athletes. Features of gaze behaviour also varied in relation to the position of the jump being approached and the round number, with the number of errors made also varying with jump number. However, the link between visual behaviour and performance success has yet to be determined. Further identification of equestrian sport-specific visual skills and their relationship with performance is now needed to enable these findings to be applied in the development of training programmes and coaching for competitive success.
